# Fatigue Crack Propagation Prediction of Corroded Steel Plate Strengthened with Carbon Fiber Reinforced Polymer (CFRP) Plates

**DOI:** 10.3390/polym14214738

**Published:** 2022-11-04

**Authors:** Anbang Li, Lu Wang, Shanhua Xu

**Affiliations:** 1 School of Civil Engineering, Xi’an University of Architecture & Technology, Xi’an 710055, China; 2 State Key Laboratory of Green Building in Western China, Xi’an University of Architecture & Technology, Xi’an 710055, China; 3 Key Laboratory of Structural Engineering and Earthquake Resistance, Ministry of Education, Xi’an 710055, China

**Keywords:** fatigue, crack propagation prediction, corroded steel plate, strengthening, carbon-fiber-reinforced polymer

## Abstract

The purpose of this study is to investigate the mechanism of improving fatigue performance and the estimation model of fatigue life for corroded steel plate strengthened with CFRP plates. A new two-stage fatigue crack propagation prediction model for the corroded steel plate strengthened with CFRP plates was proposed; moreover, the identification of critical rust pits and the equivalent method of initial cracks, and the calculation method of stress intensity factor (SIF) values at the crack tip were established. The accuracy of the proposed model was verified by comparing the predicted and tested fatigue life of the corroded steel plate strengthened with CFRP plates. Finally, the proposed two-stage crack propagation model was applied to carry out a parameter analysis to investigate the effect of weight loss rate, equivalent initial crack size, adhesive thickness, CFRP stiffness and CFRP prestress level on the fatigue crack propagation of the corroded steel plate strengthened with CFRP plates. Results showed that the maximum depth and the average width of the rust pits were suggested to be taken as the equivalent dimensions of the initial semi-elliptical surface crack for the fatigue crack propagation prediction of corroded steel plate strengthened with CFRP plates. Increasing the weight loss rate of the corroded steel plate, the initial crack size or the adhesive thickness would accelerate the crack growth and reduce the fatigue life, whereas increasing the stiffness or prestress level of the CFRP plate would significantly reduce the crack growth rate and increase the fatigue life. The smaller the initial crack size, the more sensitive the crack propagation life was to the variation of equivalent initial crack size. The influence of adhesive thickness on the fatigue life was limited and convergent, and the application of prestressing could significantly improve the utilization rate of CFRP materials and the fatigue strengthening effect of the corroded steel plate.

## 1. Introduction

Engineering practice shows that despite the application of various protective and construction measures and maintenance systems, many steel structure projects (such as electricity transmission towers, bridges, offshore production platforms, large industrial buildings, etc.), which have been exposed to long-time corrosive environments, such as ocean and industrial atmosphere environments, still find it difficult to completely avoid corrosion. Corrosion not only leads to a huge waste of resources and economic losses, but also has a serious impact on the subsequent service and safety performance of steel structures. The deterioration of fatigue performance of steel structure caused by corrosion is particularly prominent [1,2]. Carbon-fiber-reinforced polymer (CFRP) materials, which possess the significant advantages of a high strength/weight ratio, as well as excellent fatigue and corrosion resistance, have been successfully applied in architectural and civil engineering as another artificial structural material after metal and concrete in the past few decades [3,4,5]. Recently, they have become a competing alternative to the conventional strengthening and repairing materials in the field of steel structure reinforcement [6,7,8,9,10], and the strengthening system with CFRP bonded to steel substrate has been proved to be more efficient, with minimized additional permanent load, eliminated stress concentration and higher durability than the traditional repair and reinforcement methods such as welding, bolting or riveting [11,12,13]. The effectiveness of externally bonded CFRP in extending the fatigue life of corroded steel plates has been verified by the authors’ recent study [14]. Results showed that externally patching CFRP plates significantly strengthened the fatigue behavior of corroded steel plates, and even made the fatigue life of corroded steel plates recover or exceed that of the uncorroded ones. The fatigue life of the patched specimen with a strengthening stiffness ratio of about 40% even showed a fatigue life extension of more than 85.3 times with respect to the unpatched corroded steel plate, approximately two times greater than the uncorroded steel plate. This indicated that the application of externally bonded CFRP materials has excellent prospects for strengthening the fatigue performance of corroded steel structures.

The reliable application of a CFRP strengthening system in the fatigue performance improvement of corroded steel structures is dependent on the knowledge of several complex phenomena, such as the mechanism of improving fatigue performance and the estimation model of fatigue life for the corroded steel plate strengthened with CFRP materials. Several research studies have been carried out on the mechanism of improving fatigue performance of the defected steel plate strengthened with CFRP materials. The methods of digging holes, cutting notches and prefabricating cracks were usually adopted to simulate the initial defects of steel plate, which are caused by structural construction as well as fatigue load history. From the point of view of the initial defect types, the typical types of the defected steel plate strengthened with CFRP materials in the existing literature could be divided into the following categories: CFRP-strengthened steel plate with a central open hole [15], CFRP-strengthened steel plate with a central hole and two symmetric cracks at each edge of the hole [6,16,17], CFRP-strengthened steel plate with a central hole and unilateral vertical crack [18], CFRP-strengthened steel plate with two V-shaped edge notches [6,19], CFRP-strengthened steel plate with a central vertical crack [20,21], CFRP-strengthened steel plate with two U-shaped edge notches and two vertical cracks [22], CFRP-strengthened steel plate with single edge crack [23,24,25], CFRP-strengthened steel plate with a central hole and two inclined cracks [26,27], etc. The factors which have a significant influence on the fatigue strengthening effectiveness of the defected steel plates strengthened with CFRP materials may be summarized as CFRP material specification, CFRP strengthening stiffness, CFRP prestressing level, patch configuration, adhesive thickness, steel surface preparation and initial damage degree of steel plate, etc. It is generally agreed that external patching with CFRP materials not only effectively reduced the average stress amplitude of the defected steel plates, but also arrested the crack opening via a “bridging effect”, decreased the stress intensity factor and crack growth rate and consequently extended the crack propagation life of the defected steel plates.

Considering that fatigue tests were time consuming, costly and discrete, numerical analysis provides an important analytical means for studying and evaluating the fatigue performance of CFRP-strengthened defected steel structures. Researchers have carried out a series of numerical analyses on the fatigue crack propagation of the defected steel plates strengthened with CFRP materials. The common analysis process was to first calculate the stress intensity factor (SIF) at the crack tip of CFRP-strengthened steel plates with initial cracks through finite element (FE) method or boundary element (BE) method, and the crack propagation analyses were then conducted, combined with fracture mechanics theory, to calculate the fatigue propagation life of the defected steel plate strengthened with CFRP materials. Colombi et al. [16] presented a numerical analysis of the cracked steel members with a central hole and two symmetric cracks reinforced by prestress composite patch, and the SIF values in the notched plates were computed by a two-dimensional FE model in connection with the three-layer technique in order to reduce the computational effort. Zheng et al. [28] proposed a fatigue life prediction method for the CFRP-plates-strengthened steel plates with a central hole and two symmetric cracks, where the SIF values at the crack tip were calculated adopting the “brick-spring-plate” FE model. The crack propagation of the CFRP-strengthened steel plate with a central vertical crack was numerically studied by Tsouvalis et al. [20] and Wu et al. [29]; the SIF values were calculated by three-dimensional FE models. The fatigue crack growth of the single-edge notched tension specimens strengthened with different reinforcement configurations were numerically analyzed by Colombi et al. [21], the FE models were developed to evaluate the SIF values and an analytical model was proposed to predict the fatigue crack growth rate and the fatigue crack growth curves. The effect of FRP configurations on the fatigue repair effectiveness of central cracked steel plates were compared by Wang et al. [30], where the SIF values and the crack growth life of the cracked steel plates were calculated by three-dimensional linear-elastic FE models. The mode I fatigue crack arrest in tensile steel members using prestressed unbonded CFRP plates was verified by Hosseini et al. [31], where the SIF values at the fatigue precrack tip were calculated using J-integral in FE models. Compared with the FE method, the main advantage of the BE method was largely attributed to the reduction in the dimensionality of 3D problems, and only surfaces were required to be modeled and meshed to perform the required integration, resulting in substantial savings in solving time [32,33,34]. The boundary element analysis of fatigue behavior for CFRP-strengthened steel plates with central vertical crack and center inclined crack were conducted by Yu et al. [34] and Chen et al. [33], respectively. Results showed that the BE method could achieve better prediction results at less computational cost. Generally, the fatigue crack propagation of the defected steel plates strengthened with CFRP materials have been systematically investigated using numerical methods. Comparison between numerical results and experimental data demonstrated that both the FE method and the BE method were reliable for crack propagation analysis of CFRP-strengthened defected steel plate.

However, what should be pointed out is that almost all the aforementioned studies were focused on the fatigue crack propagation of CFRP-strengthened defected steel plates with initial hole, notch or cracks. The initial damage caused by corrosion, which mainly manifested as effective cross-section loss and stress concentration at rust pits, was quite different from the initial defects of digging holes, cutting notches and prefabricating cracks. The initial crack of the CFRP-strengthened defected steel plates in the existing numerical studies were generally simplified as a mode Ⅰ though crack, and the differences in the SIF values and crack propagation rate along the thickness direction of steel plate were always ignored. While the fatigue cracks of the corroded steel plate strengthened with CFRP materials initiated at the bottom of the rust pits on the steel surface, and gradually developed into partial-through surface crack and though crack with the increase in fatigue cycles [14], in addition, corrosion damage would also cause the change in the bonding performance of the interface between CFRP materials and steel substrate [35,36]. The corroded steel plate strengthened with CFRP is bound to have a distinctive mechanism and process of fatigue crack propagation with respect to the CFRP-strengthened cracked steel plates. At present, to the authors’ knowledge, there have been few numerical studies conducted to investigate the fatigue crack propagation of the corroded steel plates strengthened with CFRP materials, and there is no estimation model which can accurately predict the fatigue life of corroded steel plate strengthened with CFRP materials.

The purpose of this study is to investigate the mechanism of improving fatigue performance and the estimation model of fatigue life for the corroded steel plate strengthened with CFRP plates. The recent experimental evidence on the effectiveness of externally bonding CFRP plate to extend the fatigue life of the corroded steel plates was first summarized, and then a new two-stage fatigue crack propagation prediction model for the corroded steel plate strengthened with CFRP plates was proposed; moreover, the identification of critical rust pits and the equivalent method of initial cracks, and the calculation method of stress intensity factor (SIF) values at the crack tip were established. Furthermore, the predictive analytics of the fatigue crack propagation of corroded steel plate strengthened with CFRP plates were carried out, and comparison between prediction values and fatigue test results was adopted to verify the reliability of the fatigue crack propagation prediction. Finally, the proposed two-stage crack propagation model was applied to carry out the parameter analysis to investigate the effect of the weight loss rate of the corroded steel plate, the equivalent initial crack size, the adhesive thickness, the stiffness and prestress level of CFRP plates on the SIF values at crack tip and the fatigue crack propagation of the corroded steel plate strengthened with CFRP plates. The outcome of this study can provide meaningful references and essential data for the reliable application of a CFRP strengthening system in the fatigue performance improvement of corroded steel structures.

## 2. Experimental Evidence

To verify the effectiveness of externally bonding CFRP plates to extend the fatigue life of the corroded steel plates, the authors of this paper recently conducted a series of tests of the fatigue behavior of corroded steel plates strengthened with CFRP plates [14,37]. The corroded steel plates for the fatigue test were cut from the flanges of Q235B hot rolled H 350 × 175 × 7 × 11 beams with corrosion duration of 0, 6, 9, 15 and 18 months. Two types of unidirectional carbon-fiber-reinforced polymer (CFRP) plates, of which the cross-sections were 50 mm × 1.4 mm and 50 mm × 2.0 mm (width × thickness), respectively, were adopted. The thixotropic and solventless two-part epoxy Sikadur-30CN was selected as the structural adhesive for bonding CFRP plates to corroded steel plates. Mechanical properties of adhesive and steel plate were obtained via uniaxial tensile coupon tests based on the Chinese codes GB/T2567-2008 [38] and GB/T228.1-2010 [39], respectively. The primary mechanical properties of the materials adopted in the fatigue test were listed in Table 1.

**Figure 1:**
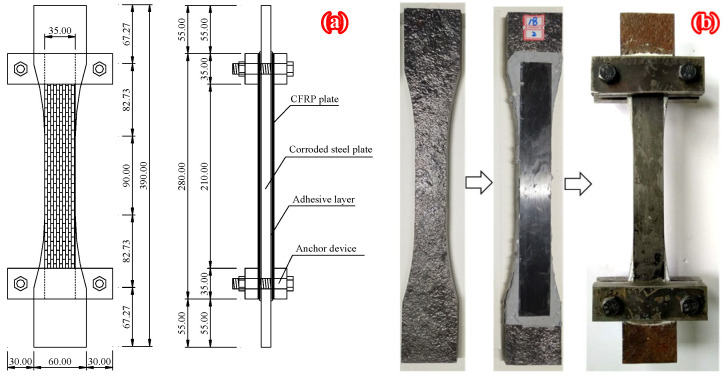
(**a**) Strengthening configuration and (**b**) manufacturing process of the corroded steel plate double-side patched with anchorage.

**Figure 2:**
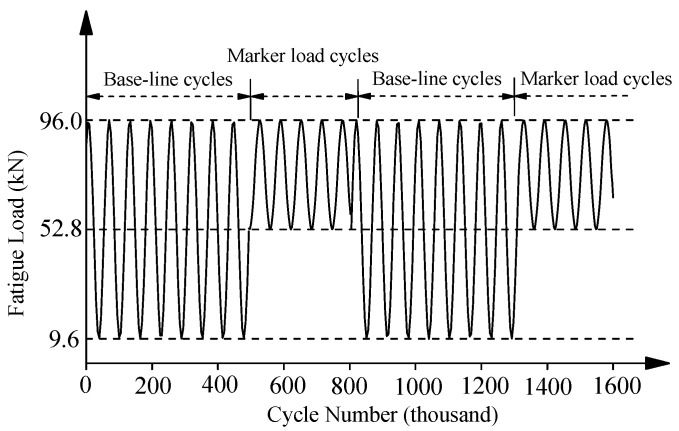
Fatigue loading cycles adopted in “beach marking” technique.

Specimens with five kinds of corrosion damage levels and four kinds of strengthening configurations were fabricated to investigate the effect of corrosion degrees and strengthening schemes on the fatigue behavior of corroded steel plates strengthened with CFRP plates. Table 2 presented a summary of specimens’ parameters, where Case A was bare steel plate without any patch, Case B was the steel plate double-side patched without anchorage, Case C was the steel plate single-side patched with anchorage and Case D was the steel plate double-side patched with anchorage. Figure 1a,b present the dimension and strengthening configuration, and the manufacturing process of the corroded steel plate that was double-sided patched with anchorage, respectively. Fatigue tests were performed on the MTS-322 electrohydraulic servo fatigue testing machine with a maximum cyclic load of ±250 kN. Specimens were tested under constant amplitude sinusoidal cyclic load with a frequency of 13 Hz, and the well-known beach-marking technique was adopted to mark the crack initiation and propagation process. Figure 2 presented the fatigue loading cycles adopted in the “beach marking” technique. As shown in Figure 2, the maximum fatigue load (P_max_) of all the strengthened specimens was 96 kN, and the load ratios (P_min_/P_max_) of baseline cycles and marker load cycles were 0.1 and 0.5, respectively. More details can be found in [14,37].

**Figure 3:**
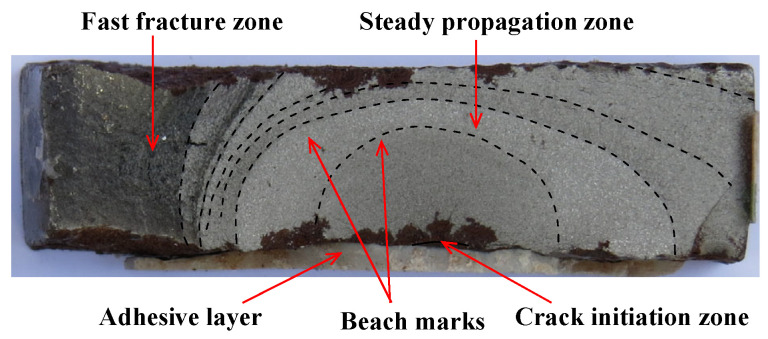
Typical fracture surface of the corroded steel plate strengthened with CFRP plates (specimen C9-DS1-A in [14,37]).

The primary fatigue behavior indications, which consist of failure mode, fatigue fractography, fatigue life and crack propagation, were analyzed. Results showed that the fatigue cracks of all the corroded steel plates strengthened with and without CFRP plates initiated at the bottom of the rust pits on the steel surface, and gradually developed into a semi-elliptical partial-through crack with the increase in load cycles. The fatigue crack propagation of corroded steel plates strengthened with CFRP plates consisted of a partial-through crack propagation stage and a through crack propagation stage; see Figure 3. Results also indicated that externally patching CFRP plates significantly strengthened the fatigue behavior of corroded steel plates, and even made the fatigue life of corroded steel plates recover or exceed that of the uncorroded ones. With respect to the corroded steel plates with the corrosion duration of 6, 9, 15 and 18 months, the weight loss rates were 9.38%, 13.39%, 16.61% and 21.24%, respectively, and the fatigue life of the strengthened specimens which were double-side patched with CFP-514 and mechanical anchorage at the end of the CFRP plates was extended by factors of 18.4, 9.3, 8.9 and 11.4 over the unpatched ones, respectively. Different strengthening configurations appeared to have diverse effects on the fatigue life extension and crack propagation retardation of corroded steel plates. End anchorage effectively delayed the failure process of the CFRP bonding interface and improved the fatigue strengthening effectiveness. Increasing the CFRP strengthening stiffness or adopting prestressed CFRP plates significantly reduced the crack propagation rate and extended the fatigue life of corroded steel plates.

## 3. Two-Stage Crack Propagation Analysis Model of Corroded Steel Plate Strengthened with CFRP Plates

**Figure 4:**
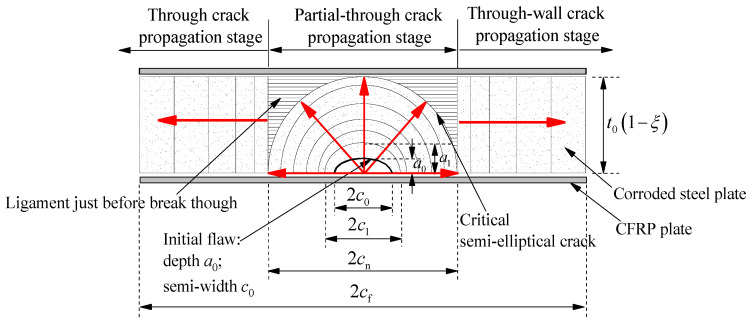
Two-stage crack propagation analysis model of corroded steel plate strengthened with CFRP plates (where $2c_{0}$ and $a_{0}$ are the width and depth of the initial surface crack, respectively, $t_{0}$ is the thickness of the un-corroded steel plate, *ξ* is the weight loss rate of the corroded steel plate, $2c_{n}$ and $2c_{f}$ are the corresponding crack width at the end of partial-though crack propagation stage and though crack propagation stage, respectively).

As shown in Figure 3, the crack propagation of the corroded steel plate strengthened with CFRP plates is a three-dimensional problem. The fatigue crack initiated and propagated from the bottom of the rust pits on the steel surface, and gradually developed from a semi elliptical partial-through crack to a penetration crack break through the thickness of the corroded steel plate. The crack propagation process of the corroded steel plate strengthened with CFRP plates was affected by many factors, such as the weight loss rate of corroded steel plate, the shape, size and position of the critical rust pits, the strengthening configuration of CFRP plates, the load transfer path of interface between the CFRP plate and corroded steel plate, etc. The stress intensity factor (SIF) and crack growth rate at different points along the crack front are completely different during the propagation process. To simplify the analysis process of crack propagation, in this study, the whole process of crack propagation of corroded steel plate strengthened with CFRP plates was divided into two stages; see Figure 4. The two-stage crack propagation prediction model of the corroded steel plate strengthened with CFRP plates was then proposed, and four basic characteristics of the model can be expressed as follows:

(1) The fatigue crack propagation process of the corroded steel plate strengthened with CFRP plates consists of the partial-through crack propagation stage and the through crack propagation stage.
(2) The critical pit is equivalent to an initial semi-elliptical surface crack, and the shape of a growing crack remains semi-elliptical during the partial-through crack propagation stage.
(3) Ignoring the rapid propagation process of the ligament just before break-though, the critical semi-elliptical crack at the end of the partial-though crack propagation stage is equivalent to a though-wall crack with the same width and is taken as the initial crack at the though crack propagation stage.
(4) In the stage of through crack propagation, the difference in SIF along the thickness direction of the steel plate is ignored, and the crack propagation rate is calculated by taking the SIF of the crack tip in the middle of the thickness of the steel plate.

Linear elastic fracture mechanics concepts are used to investigate the fatigue crack growth. According to Paris [40], the relation between the crack growth rate da/dN and the SIF amplitude ΔK in the stable growth region can be described by a power function:

(1) $$\frac{da}{dN}=C\Delta K^{m}$$

with *C* and the exponent *m* as material constants. The equation in a double log plot gives a linear relation: log(da/dN)=log(C)+mlog(ΔK) with *m* as the slope of the linear function.

**Figure 5:**
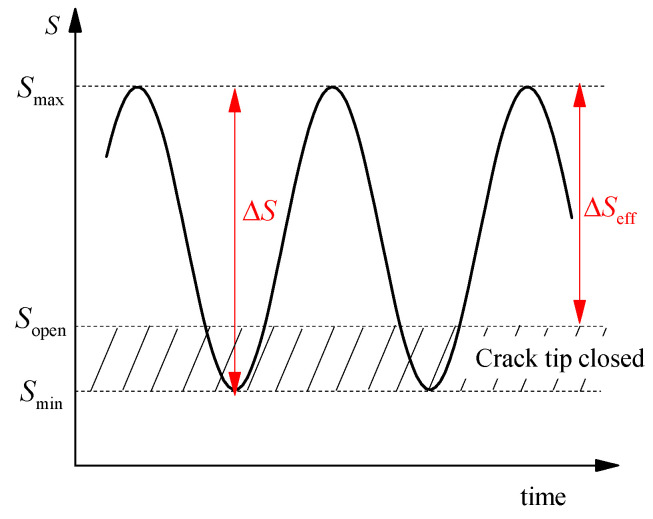
Plasticity-induced crack closure (where $S_{\max}$ and $S_{\min}$ are the maximum and minimum stress, $S_{\mathrm{open}}$ is the minimum tension stress corresponding to crack opening).

Elber [41] discovered that the fatigue crack under the tensile load was already closed during unloading before the tension stress became zero via fatigue test, which was called the plasticity-induced crack closure phenomenon. According to the concept of SIF, the stress singularity at the tip of the crack, defined by the SIF, is present as long as the crack tip is open. Elber proposed that the stress variation will contribute to crack extension only if the stress singularity occurs at the crack tip, which drew out a new concept; namely, the effective stress range $\Delta S_{\mathrm{eff}}$, as shown in Figure 4. The corresponding driving force of crack propagation became the effective SIF amplitude $\Delta K_{\mathrm{eff}}$:

(2) $$\frac{da}{dN}=C\Delta K_{\mathrm{eff}}^{m}$$

where $\Delta K_{\mathrm{eff}}=K_{\max}-K_{\mathrm{open}}$, $K_{\max}$ and $K_{\mathrm{open}}$ were SIF values corresponding to $S_{\max}$ and $S_{\mathrm{open}}$, respectively, as shown in Figure 5.

Elber defines the ratio *U*:

(3) $$U=\frac{\Delta K_{\mathrm{eff}}}{\Delta K}$$

Substituting Equation (3) into Equation (2) yields

(4) $$\frac{da}{dN}=C{\left(U\Delta K\right)}^{m}$$

It was found that the ratio *U* depended on the type of material and the stress ratio *R*. As for the low-carbon steel (Q235) involved in this study, the ratio *U* could be calculated by adopting the following equation [42]:

(5) U=0.69+0.45R

According to the similarity principle of crack propagation, i.e., Equation (4), the growth increment of the cracks in the depth and width directions during the partial-though crack propagation stage, as shown in Figure 4, shall meet the following relationships at the same time.

(6) $$\Delta a_{i}=a_{i+1}-a_{i}=\Delta N_{i}\cdot C{\left(U\Delta K_{a_{i}}\right)}^{m}$$

(7) $$\Delta c_{i}=c_{i+1}-c_{i}=\Delta N_{i}\cdot C{\left(U\Delta K_{c_{i}}\right)}^{m}$$

where $\Delta a_{i}$ and $\Delta c_{i}$ were the growth increments of the cracks in the depth and width directions, respectively; ΔN was the increment of fatigue cycles, Δ*Ka_i_* and $\Delta K_{c_{i}}$ and were the stress intensity factor amplitudes corresponding to the position of crack depth $a_{i}$ and half-width $c_{i}$, respectively. It can be obtained from Equations (6) and (7):

(8) $$\frac{\Delta a_{i}}{\Delta c_{i}}={\left(\frac{\Delta K_{a_{i}}}{\Delta K_{c_{i}}}\right)}^{m}$$

It can be found from Equation (8) that the ratio of the crack growth increment in the depth and width directions for the semi-elliptic partial-though crack is a power function relationship with the ratio of the corresponding SIF amplitude. When the crack depth growth increment is $\Delta a_{i}$, the corresponding number of fatigue cycles Δ*N_i_* and the crack width growth increment $\Delta c_{i}$ can be expressed as:

(9) $$\Delta N_{i}=\frac{\Delta a_{i}}{C{\left(U\Delta K_{a_{i}}\right)}^{m}}$$

(10) $$\Delta c_{i}=\Delta a_{i}{\left(\frac{\Delta K_{c_{i}}}{\Delta K_{a_{i}}}\right)}^{m}$$

Then, the new size of the semi-elliptical crack is obtained:

(11) $$a_{i+1}=a_{i}+\Delta a_{i}$$

(12) $$c_{i+1}=c_{i}+\Delta c_{i}$$

Recalculate the corresponding SIF amplitude based on the new size of the semi-elliptic crack. Repeat the above process until the crack depth reaches the thickness value of the steel plate, and the partial-though crack propagation stage is ended. The cumulative crack propagation life $N_{1}$ and the width of the corresponding critical semi-elliptical crack $c_{n}$ at the end of this stage are obtained:

(13) $$N_{1}=\sum_{i=0}^{n}\Delta N_{i}=\sum_{i=0}^{n}\frac{\Delta a_{i}}{C{\left(U\Delta K_{a_{i}}\right)}^{m}}$$

(14) $$c_{n}=c_{0}+\sum_{i=0}^{n}\Delta c_{i}=c_{0}+\sum_{i=0}^{n}\Delta a_{i}{\left(\frac{\Delta K_{c_{i}}}{\Delta K_{a_{i}}}\right)}^{m}$$

During the though crack propagation stage, the width of the corresponding critical semi-elliptical crack $c_{n}$ is taken as the initial crack size ($c_{j}=c_{n}$) to calculate the SIF amplitude $\Delta K_{c_{j}}$ at the crack front. The crack growth increment of the through crack propagation stage is defined as $\Delta c_{j}$, and the corresponding crack growth life can be expressed as:

(15) $$\Delta N_{j}=\frac{\Delta c_{j}}{C{\left(U\Delta K_{c_{j}}\right)}^{m}}$$

Accordingly, the new size of the though crack is obtained,

(16) $$c_{j+1}=c_{j}+\Delta c_{j}$$

Recalculate the corresponding SIF amplitude $\Delta K_{c_{j+1}}$ and repeat the above process until the SIF of the through crack reaches the limit value of the stable tearing crack growth region $K_{c}$, and the specimen is fractured. The cumulative crack propagation life $N_{2}$ of the though crack propagation stage can be expressed as:

(17) $$N_{2}=\sum_{j=n}^{k}\Delta N_{j}=\sum_{j=n}^{k}\frac{\Delta c_{j}}{C{\left(U\Delta K_{c_{j}}\right)}^{m}}$$

Consequently, the fatigue life of the whole process of crack propagation can be expressed as follows:

(18) $$N=N_{1}+N_{2}=\sum_{i=0}^{n}\frac{\Delta a_{i}}{C{\left(U\Delta K_{a_{i}}\right)}^{m}}+\sum_{j=n}^{k}\frac{\Delta c_{j}}{C{\left(U\Delta K_{c_{j}}\right)}^{m}}$$

The above two-stage crack propagation analysis is a continuous iterative calculation process. To ensure the precision, the crack growth increments $\Delta a_{i}$ and $\Delta c_{j}$ adopted for each calculation step should be small enough to ensure that the change in crack growth rate within the corresponding crack growth increment can be ignored. In addition, according to the above two-stage crack propagation model, the following information is essential for the fatigue crack propagation analysis of the corroded steel plate strengthened with CFRP plates: ① The driving force of crack propagation, i.e., the *K*-values at crack tip along the crack front which were related to the dimensions of the initial cracks, the weight loss rate of the corroded steel plate, the stiffness and prestress level of the CFRP plates, etc. ② Crack propagation resistance of the material, i.e., the calibration values of the material constants *C* and *m* in Equation (4) for the corroded steel plate. ③ Equivalent initial crack size, i.e., the identification of critical rust pits on the corroded steel surface and the equivalent method of the initial cracks. Information on the above three aspects will be provided hereinafter.

## 4. Material Constant Calibration

The material constants *C* and *m* in Equation (4) can be calibrated by the relationship between the crack growth rate da/dN and the SIF amplitude ΔK. Taking the logarithm on both sides of Equation (4) yields

(19) lg(da/dN)=lgC+mlog(UΔK)

where da/dN can be obtained by means of a crack propagation test and approximate calculation of secant method or fitting method, and ΔK can be obtained by finite element calculation or consulting the manual of SIF values. The crack propagation data of uncorroded Q235 steel plate with a thickness of 10 mm and a stress ratio of 0.1 were determined with single-edge notched three-point bending specimens by Dong and Mei [43]. Wang [44] conducted an experimental study to investigate the effect of corrosion on the fatigue life and crack growth rate of low-carbon steel plates. The electrochemical fast corrosion test method was adopted to acquire corroded steel plate, and the crack propagation data of the corroded center cracked tension specimen with a thickness of 3 mm and stress ratio of 0.2 were obtained. Herein, the material constants *C* and *m* are calibrated by using the crack propagation test data reported in the aforementioned literatures, combined with the calculated values of the corresponding SIF.

**Figure 6:**
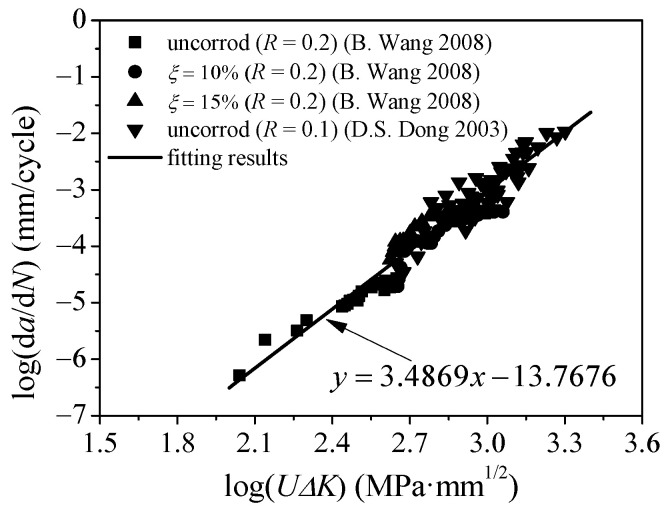
Relationship between the crack propagation rate and the effective SIF amplitude of Q235 steel plates [43,44].

Figure 6 illustrates the relationship between the crack propagation rate and the effective SIF amplitude of Q235 steel plates. As shown in Figure 6, the crack propagation rate presented a good linear relationship with the effective stress intensity amplitude under the double logarithmic coordinate system, and the variation in corrosion rate *ξ* has little influence on the fatigue crack propagation rate. The main reason for this may be that the local stress concentration caused by rust pits on the corroded steel surface could be ignored compared with the stress singularity at the crack tip. The data in Figure 6 are fitted using the least square method, and the optimum fit values of the material constants *C* and *m* of the Q235 corroded steel plates are 1.7075 × 10^−14^ and 3.4869, respectively.

## 5. Identification of Critical Rust Pits and the Equivalent of Initial Cracks

Through the fatigue fracture analysis in the authors’ recent study [14], it could be found that the fatigue cracks of the corroded steel plate strengthened with CFRP plate initiated at the bottom of the rust pit on the surface of the corroded steel plate, and almost all of them originated from a single crack source. In practice, the fatigue crack propagation analysis of the corroded components was also generally regarded as a single crack propagation process, and the rust pits were converted into an equivalent initial flaw according to specific equivalence rules, and then the fatigue crack propagation prediction was conducted based on the classical fracture mechanics theory [45,46,47]. In this study, the same idea was adopted for the fatigue crack propagation prediction of the corroded steel plates strengthened with CFRP plates and, accordingly, the identification of the critical rust pits and the equivalent of the initial cracks became the basis for the fatigue crack propagation prediction of the corroded steel plate strengthened with CFRP plates. To the authors’ knowledge, however, at present, researchers have not formed a unified understanding of the identification and equivalent of the critical rust pits. Dolley et al. [45] equated the rust pit to a semi-circular surface crack according to the maximum pit depth and carried out a crack propagation analysis on the corroded 2024-T3 aluminum alloy plate. It was found that the crack depth has a significant correlation with the distribution characteristics of fatigue life. Duquesnay et al. [47] equated the rust pit to a semi-elliptical surface crack and took the maximum pit depth and the average pit width as the equivalent dimension of the initial crack to predict the crack propagation life of the rust-damaged 7075-T6511 aluminum alloy plate. Sankaran et al. [46] equated the average depth and width of the corrosion pit to a semi-elliptical surface crack and predicted the crack propagation life of corroded 7075-T6 aluminum alloy, which also achieved good results. Zhang and Yao [48] advocated that the opening angle at the bottom of rust pit could be used to determine the critical corrosion pit. It was found that the fatigue life of the pre-corroded LC4CS aluminum plate could be predicted with reasonable accuracy by using the opening angle to determine the critical corrosion pit and converting the critical corrosion pit to the initial crack with the same depth and width. Xu and Wang [49] determined the position of the critical corrosion pit through surface topography measurements and stress concentration analysis, and equated the corrosion pit to semi-elliptical surface crack, and then predicted the fatigue life of corroded steel plate, which was in good agreement with the test results.

Corrosion damage of the steel plate consists of uniform corrosion and pitting corrosion. The uniform corrosion mainly causes a reduction in the thickness and the effective cross-sectional area of the corroded steel plate, which leads to an increase in the average cross-sectional stress of the corroded steel structure. The pitting corrosion and other local damages mainly cause the stress concentration on the surface of steel plate and promote crack initiation and propagation. The research results of the surface morphology characteristics of the corroded steel plates, which were presented in the authors’ recent study [35], showed that with the increase in the corrosion duration, the corrosion pits grew alternately in the depth and width direction. Local pitting corrosion and uniform corrosion alternately played the dominant role in the surface topography and roughness of corroded steel plates, and the statistical characteristic parameters of the corrosion surface morphology fluctuated periodically.

**Figure 7:**
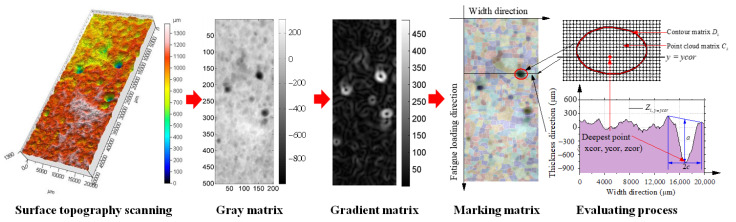
Identification, extraction and evaluation process of rust pits on the surface of corroded steel plate.

Figure 7 illustrates a method for identifying, extracting and evaluating rust pits on the surface of corroded steel plates based on three-dimensional topography scanning and watershed algorithm. As shown in Figure 7, the three-dimensional point cloud data were obtained via surface topography scanning of the corroded steel plates, and then the surface topography data of the corroded steel plate were directly written into the gray matrix through coordinate transformation to avoid the loss of surface corrosion details. Then, the gray matrix was successively filtered, and the gradient and watershed were calculated to obtain the marking matrix of the rust pits. Finally, the point cloud matrix and contour matrix of each rust pit were obtained by extracting the specific elements of the marking matrix. The dimensions (i.e., depth and width) of the critical rust pits perpendicular to the fatigue load direction could be calculated via the following equations:

(20) $$a=\frac{1}{2}\left(\max(D_{k}{\left(:,3\right)}_{y=ycor})+\min(D_{k}{\left(:,3\right)}_{y=ycor})\right)-\min(C_{k}\left(:,3)\right)$$

(21) $$c=\frac{1}{2}\left(\max(D_{k}{\left(:,1\right)}_{y=ycor})+\min(D_{k}{\left(:,1\right)}_{y=ycor})\right)$$

where *C_k_* was the point cloud matrix (*m* rows × 3 columns) of the *k*-th rust pit, *m* represented the total number of scanning points within the range of the rust pit and the three columns store the *X_i_*, *Y_i_* and *Z_i_* coordinates of the scanning points within the range of the rust pit; *D_k_* was the contour matrix of the *k*-th rust pit (*n* rows × three columns), *n* was the number of scanning points of the contour of *k*-th rust pit and three columns stored the *X_i_*, *Y_i_* and *Z_i_* coordinates of the scanning points. *y* = *ycor* represents the section plane along the width direction, passing through the local deepest point in the k-th rust pit, as shown in Figure 7. More details about the aforementioned calculation process can be found in the authors’ recent study [37].

The statistical results of the size and shape characteristics of the rust pits on the surface of the corroded steel plates showed that the volume ratio of the “critical rust pits” (i.e., the deep rust pits), which present as the ratio of the volume of the rust pit to the volume of the smallest cuboid surrounding the rust pit, was mainly distributed around π/6 [37], which indicated that the shape of most deep pits was similar to that of semi (ellipsoid) spheres. Consequently, the following equivalent rules were proposed for the initial crack of corroded steel plate strengthened with CFRP plates based on the crack initiation mechanism at the rust pit and referring to the existing research results:

(1) The thickness of the corroded steel plate is converted according to the weight loss rate *ξ* and the thickness of the uncorroded steel plate, and the critical corrosion pit is equivalent to a semi-elliptical surface crack located at the center of the corroded steel plate.
(2) The dimension (i.e., the depth and width) of the equivalent initial semi-elliptical surface crack includes two kinds of constitution, where the width and depth of the critical rust pit (i.e., the deepest rust pit) are taken as the equivalent dimension of type I crack, the maximum depth and the average width of the rust pits are taken as the equivalent dimension of type II crack.
(3) The depth and width of the rust pit are calculated according to Equations (20) and (21), and the calculation process of the dimension of all corrosion pits on the corroded steel surface is carried out by adopting the developed rust pit identification, extraction and evaluation program.

## 6. Calculation Method of SIF at Crack Tip of Corroded Steel Plate Strengthened with CFRP Plates

The crack initiated and propagated from the critical corrosion pit position, and gradually developed from a partial-though crack to a penetration crack through the thickness of the steel plate. Finally, the residual net section of the corroded steel plate was insufficient to bear the external load, resulting in the fatigue fracture failure of the specimen. During this process, the crack front of the corroded steel plate strengthened with CFRP plates was curved, and the fatigue crack propagation problem had a 3D character. The stress intensity factors and the crack propagation rate at each point along the crack front were different. Obviously, the crack propagation process of corroded steel plate strengthened with CFRP plates was significantly affected by many factors, such as the weight loss rate of corroded steel plate, the equivalent dimension of the initial crack (the dimension of the critical rust pit), the reinforcement parameters of CFRP plates (the thickness of the adhesive layer, the stiffness and prestress level of the CFRP plate, etc.). The calculation method of the SIF at crack tip is the basis of predicting crack propagation of corroded steel plate strengthened with CFRP plates.

The calculation methods of SIF include the analytical method and the numerical method, where the analytical method includes the stress function method and integral transformation method, the numerical method includes the finite element method, the boundary element method, the boundary collocation method, etc. The *K*-value at each point along the crack front for the corroded steel plate strengthened with CFRP plate can be derived by calculating the strain energy release rate *G* at each point along the crack front and combining the relationship between the energy release rate and the SIF. In this study, the crack opening modes of the corroded steel plate strengthened with CFRP plate is opening in tension, which belongs to Mode I crack, and the following relationship is satisfied for mode I crack:

(22) $$K_{I}=\sqrt{G_{I}E}$$

where $K_{I}$ is the SIF value of the mode I crack, *E* is the tensile modulus of elasticity, and $G_{I}$ is the energy release rate of mode I crack which could be obtained by finite element method.

### 6.1. Finite Element Model

**Figure 8:**
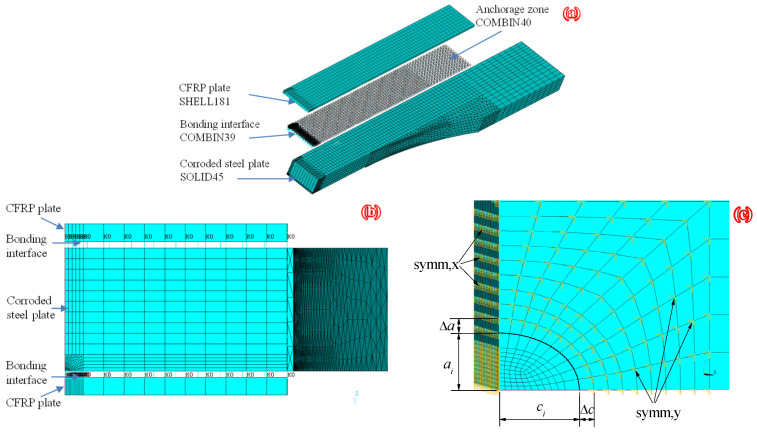
“Brick-Spring-Plate” model: (**a**) Schematic diagram of element selection and division, (**b**) fatigue crack propagation surface in FEM, and (**c**) element division near the crack front in the partial-through crack stage (where $a_{i}$ and $c_{i}$ are the depth and half-width of the crack, respectively, Δa and Δc are the growth increments of the cracks in the depth and width directions, respectively).

The specimen dimensions and configuration applied in the authors’ recent experimental study [14], and shown in Figure 1, were adopted as the prototype structure. The commercial finite element code (ANSYS^®^ 14.5) was employed, and the finite element model (FEM) of “Brick-Spring-Plate” was established to investigate the nodal force and opening displacement of elements before and after crack front of corroded steel plate strengthened with CFRP plates. Due to the existing symmetry, one quarter of the patched specimen was modeled by taking symmetric structures along the width and length of the specimen with the FE method, as shown in Figure 8. The corroded steel plate and the CFRP plate are simulated by eight-node solid element solid45 and four-node finite strain shell element shell181, respectively. The adhesive layer between CFRP plate and corroded steel surface is simulated by the nonlinear spring element COMBIN39; that is, three mutually perpendicular spring elements are inserted between the element nodes of corroded steel plate and CFRP plate, and the degree of freedom constraints in the x, y and z directions are realized by rewriting the element characteristic parameters KEYPOT (4). The static friction force of CFRP plate in the anchorage zone is simulated by bilinear force-deformation element COMBIN40. The load transfer and failure process of the interface between CFRP plate and steel plate in the anchorage zone are simulated by the “separation” function of COMBIN40.

The purpose of finite element analysis is to calculate the strain energy release rate at the crack tip, so as to calculate the SIF at the crack tip based on Equation (22). Considering that SIF is an elastic concept, the material model for the corroded steel plate is assumed to be linearly elastic. The elastic modulus and Poisson’s ratio of steel plate were 181.9 GPa and 0.3, respectively, and the thickness of corroded steel plate was $t_{s}=t_{0}\left(1-\xi \right)$, where $t_{0}$ was the thickness of uncorroded steel plate and *ξ* was the weight loss rate of the corroded steel plate. The line elastic constitutive model was adopted for the CFRP plate with an elastic modulus of 165 GPa and Poisson’s ratio of 0.3. The real constant of the spring element COMBIN39 (i.e., the *F*-*D* curves), which reflect the bond–slip relationship of the bonding interface between CFRP plate and corroded steel plate, was calculated according to the interfacial bond–slip model of CFRP plate externally bonded to corroded steel plate reported in the authors’ recent study [35,36,50]:

(23) $$F=\tau a^{2},D=s$$

where *F* was the force of the spring element COMBIN39, *D* was the displacement between the nodes at both ends of the spring element, *τ* and *s* were the interfacial shear stress and relative slip, respectively, and $a^{2}$ was the area covered by individual springs. The real constant (FSLIDE) of the bilinear force-deformation element COMBIN40 was calculated according to the maximum static friction force of the CFRP plate in the anchorage zone:

(24) $$FSLIDE=2\mu P_{\max}a^{2}/A_{\mathrm{anch}}$$

where $P_{\max}$ is the tensile force of the high-strength bolt in the anchor device (see Figure 1, and more details can be found in [14]); μ is the static friction coefficient between CFRP plate and steel plate; $A_{\mathrm{anch}}$ is the compression area of CFRP plate in the anchorage zone. The prestress of the CFRP plate was achieved by applying temperature load to the CFRP plate, and the temperature load could be calculated using the following equation:

(25) $$\Delta T=\alpha f_{u}/\left(E_{c}\delta_{c}\right)$$

where α was the prestress level, defined as the ratio of the prestress to the tensile strength of the CFRP plate; $f_{u}$ and $E_{c}$ were the ultimate tensile strength and elastic modulus of CFRP plate, respectively; $\delta_{c}$ was the coefficient of linear expansion of temperature of CFRP plate, $\delta_{c}$ = 1 × 10^−5^/°C.

### 6.2. Calculation Method of Strain Energy Release Rate

**Figure 9:**
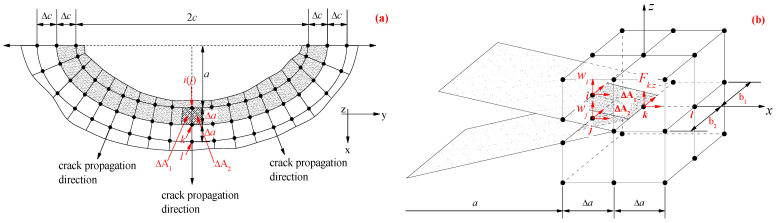
Schematic diagram of calculating strain energy release rate of surface crack by virtual crack closure technique: (**a**) Propagation section of semi-elliptical surface crack, (**b**) sketch map of the nodal force in front of the crack propagation and the opening displacements at the end of the crack propagation.(where 2c and a are the width and depth of the surface crack, respectively; Δa and Δc are the virtual crack growth increment in the depth and width directions, respectively; $F_{k,z}$ is the nodal force of the element in front of the crack propagation in the normal direction of the crack propagation surface; $w_{i}$ and $w_{j}$ are the opening displacements of node *i* and node *j* in the normal direction of the crack growth surface, respectively; $\Delta A_{1}$ and $\Delta A_{2}$ are the area of the opening crack during the virtual crack propagation, $b_{1}$ and $b_{2}$ are the width of elements on both sides of the crack propagation direction, respectively).

Through the above finite element analysis, the nodal force and opening displacement of elements before and after crack front are obtained, and then the strain energy release rate $G_{I}$ at crack tip can be calculated using a virtual crack closure technique. According to Griffith’s energy release theory, the energy released by the crack tip is equal to the work conducted by the upper and lower surfaces of the crack when the crack is propagated. Figure 9 presents a schematic diagram of calculating strain energy release rate of surface crack by virtual crack closure technique. As shown in Figure 9, it is considered from virtual crack closure technique that with respect to the crack with a length of *a*, when it propagates from a+Δa (node *k*) to a+2Δa (node *l*) with an increment of Δa, the energy released by the crack tip ΔE is equal to the energy required to close the crack from node *k* to node *l*. Moreover, it is also assumed from virtual crack closure technique that the stress and displacement of the node at the crack tip will not be significantly changed during the crack propagation process from a+Δa (node *k*) to a+2Δa (node *l*) if the increment of Δa is considerably small. According to the above assumptions, the work conducted during the crack growth process on the upper and lower surface of the crack, i.e., the energy released by the crack tip ΔE, can be calculated using the node force of the element in front of the crack propagation and the opening displacement at the end of crack propagation. In this study, the crack opening mode of the corroded steel plate strengthened with CFRP plate belongs to mode I crack, and the corresponding strain energy release rate $G_{I}$ can be calculated according to the following Equation [51]:

(26) $$G_{I}=\frac{\Delta E}{2\left(\Delta A_{1}+\Delta A_{2}\right)}=\frac{F_{k,z}\left(w_{i}-w_{j}\right)}{2\left(\Delta A_{1}+\Delta A_{2}\right)}$$

where $F_{k,z}$ is the nodal force of the element in front of the crack propagation in the normal direction of the crack propagation surface; $w_{i}$ and $w_{j}$ are the opening displacements of node *i* and node *j* in the normal direction of the crack growth surface, respectively; $\Delta A_{1}+\Delta A_{2}$ is the area of the opening crack during the virtual crack propagation, $\Delta A_{1}=\frac{1}{2}\Delta a\cdot b_{1}$, $\Delta A_{2}=\frac{1}{2}\Delta a\cdot b_{2}$, Δa is the virtual crack growth increment, $b_{1}$ and $b_{2}$ are the width of elements on both sides of the crack propagation direction, respectively, as shown in Figure 9. Consequently, the SIF at the crack tip can be calculated by substituting Equation (26) into Equation (22).

According to the analysis results of the aforementioned “Brick-Spring-Plate” model and the calculation method of strain energy release rate presented in Section 6.1 and Section 6.2, respectively, the SIF at any position along the crack front can be obtained, and then the fatigue crack propagation prediction of the corroded steel plate strengthened with CFRP plates can be executed.

## 7. Case Analysis and Model Verification

### 7.1. Case Analysis

The two-stage crack propagation model, the equivalent method of initial cracks and the calculation method of SIF at the crack tip of corroded steel plate strengthened with CFRP plates were proposed in the previous text. In this section, the specimen dimensions and configuration involved in the fatigue test in the authors’ recent study [14] were adopted as the prototype structure, and the crack propagation analysis was carried out as a case study to illustrate the whole process of fatigue crack propagation prediction of the corroded steel plate strengthened with CFRP plates. The distribution characteristics of the SIF along the crack front for the corroded steel plate strengthened with CFRP plates with respect to the crack propagation were also investigated. Taking the specimen C9-DS1-A applied in [14] as an example, the specimen was double-side patched with CFP-514 with a thickness of 1.4 mm and a width of 35 mm, and the anchorage device was installed at both ends of the specimen. The thickness of the adhesive layer of the strengthened specimens was 0.5 mm. The weight loss rate and the converted thickness of the corroded steel plate were 13.25% and 9.326 mm, respectively. The dimensions of the equivalent initial crack were as follows: $a_{0}$ = 0.51 mm, $c_{0}$ = 0.68 mm. The whole process of the fatigue crack propagation prediction of specimen C9-DS1-A is listed in Table 3. As shown in Table 3, the prediction method is a continuous iterative calculation process, and the crack size adopted in the *n*-th step ($a_{n}$ and $c_{n}$) is the sum of the crack size used in the (*n* − 1)-th step ($a_{n-1}$ and $c_{n-1}$) and the crack growth increment obtained in (*n* − 1)-th step ($\Delta a_{n-1}$ and $\Delta c_{n-1}$). Step 1~step 21 in Table 3 belong to the partial-through crack propagation stage, and step 22~step 30 belong to the through crack propagation stage.

**Figure 10:**
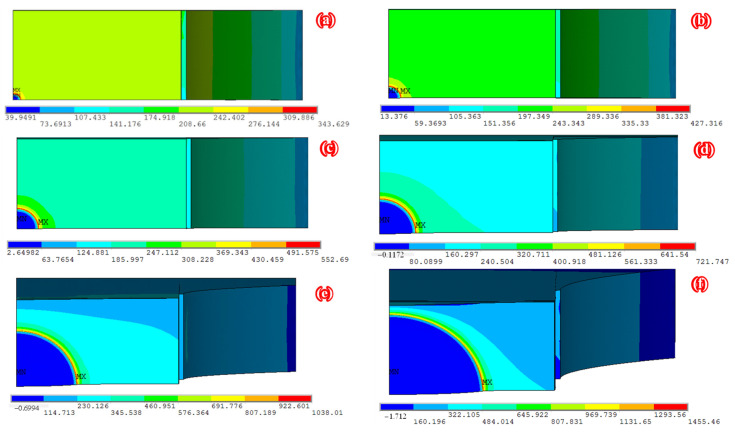
Distribution characteristic of tensile stress along the crack front of the semi-elliptical surface crack of specimen C9-DS1-A during partial-through crack propagation stage (unit: MPa): (**a**) step 1, (**b**) step 3, (**c**) step 6, (**d**) step 10, (**e**) step 15 and (**f**) step 20.

Figure 10 presents the distribution characteristic of tensile stress along the crack front of the semi-elliptical surface crack of specimen C9-DS1-A during the partial-through crack propagation stage. As shown in Figure 10, with the continuous crack propagation, the tensile stress at each point along the crack front increases continuously, and the position of the maximum tensile stress gradually shifts from the deepest point to the widest point of the semi-elliptical surface crack.

**Figure 11:**
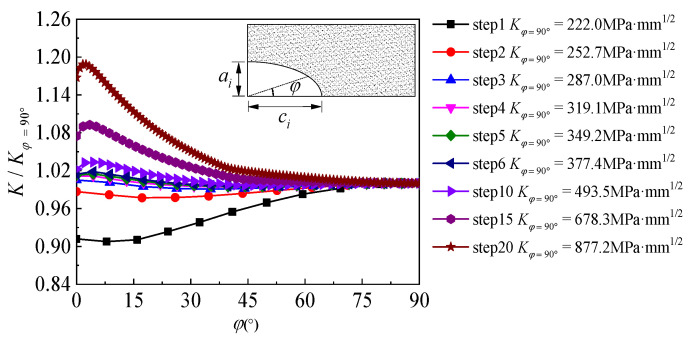
Distribution characteristics of SIF along the crack front of the semi-elliptical surface crack of specimen C9-DS1-A during partial-through crack propagation stage.

Figure 11 illustrates the distribution characteristic of SIF along the crack front of the semi-elliptical surface crack of specimen C9-DS1-A during the partial-through crack propagation stage, where the abscissa is the location angle *φ* which represents the position along the semi-elliptic crack front and the ordinate is the ratio of the SIF at each point along the crack front to the SIF at the deepest point ($\varphi =90^{\circ }$). As illustrated in Figure 11, at the initial stage of crack propagation, the *K*-value at the deepest point ($\varphi =90^{\circ }$) is significantly larger than that at the material surface ($\varphi =0^{\circ }$), which means that the crack propagation rate in the depth direction will then be faster than that along the width direction, and the “slender” initial crack will become less “slender” with the continuous crack propagation. As the crack continues to propagate, the ratio of the SIF at each point along the crack front to the SIF at the deepest point gradually shrinks to approximately 1.0, the distribution of the SIF and crack growth rate at each point along the crack front tends to be gradual and consistent (see step 4~step 6 in Figure 11) and the shape characteristic (i.e., the ratio of width to depth) of the crack front tends to be stable. As the crack propagation continues, when the crack front is about to penetrate the thickness of the steel plate (see step 15~step 20 in Figure 11), the SIF values at each point along the crack front present significant differences again. The position of the maximum SIF shifts from the deepest point of the crack to the material surface, which means that the crack propagation rate near the steel plate surface will be faster than that along the crack depth direction before penetration, and the shape of the crack, i.e., the ratio of width to depth, becomes “slender” again.

**Figure 12:**
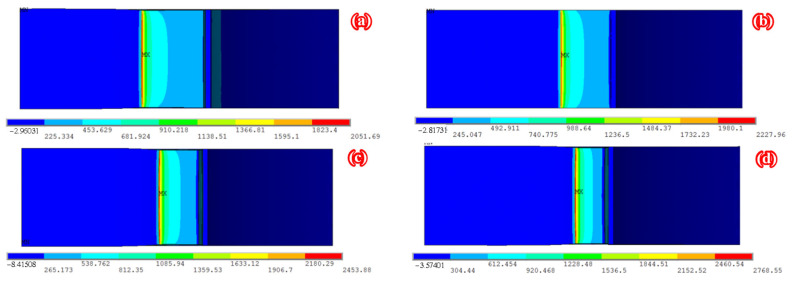
Distribution of tensile stress along the crack front of specimen C9-DS1-A during through crack propagation stage (unit: MPa): (**a**) step 22, (**b**) step 24, (**c**) step 26, and (**d**) step 28.

**Figure 13:**
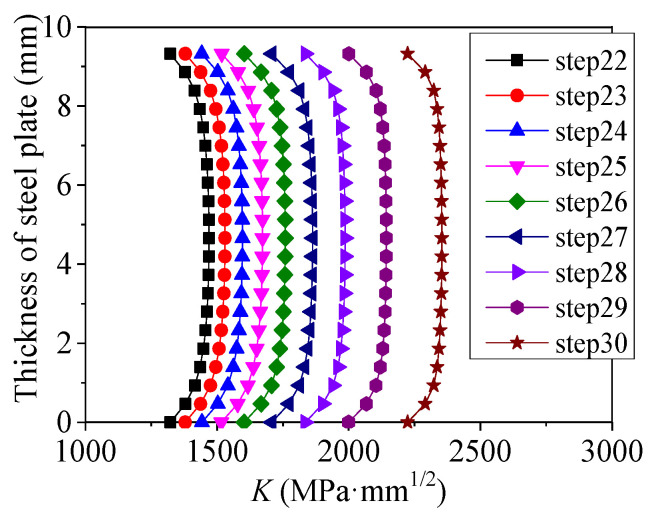
Distribution characteristics of SIF at crack front of specimen C9-DS1-A during through crack propagation stage.

Figure 12 shows the distribution of tensile stress along the crack front of specimen C9-DS1-A during the through crack propagation stage. As shown in Figure 12, the tensile stress at each point along the crack front increases continuously with the continuous crack propagation; moreover, the tensile stress is symmetrically distributed along the thickness direction of the steel plate and the maximum tensile stress is in the middle of the plate thickness. Figure 13 depicts the distribution characteristic of the SIF at the crack front along the thickness direction during the though crack propagation stage. It can be found from Figure 13 that the SIF values at the crack front are symmetrically distributed along the thickness direction of the steel plate. The *K*-value at the crack front in the middle of the steel plate thickness is greater than that at the surface of the steel plate, which means that the crack propagation rate in the middle of the steel plate thickness is greater than that at the surface of the steel plate. This phenomenon is consistent with the difference in the crack growth rate between the material surface and the middle of the steel plate thickness caused by the plastic induced crack closure effect, and it also explains the phenomenon that the crack front gradually protrudes forward and outward with the crack propagation, which was observed in the authors’ recent fatigue test [14].

### 7.2. Model Verification

**Figure 14:**
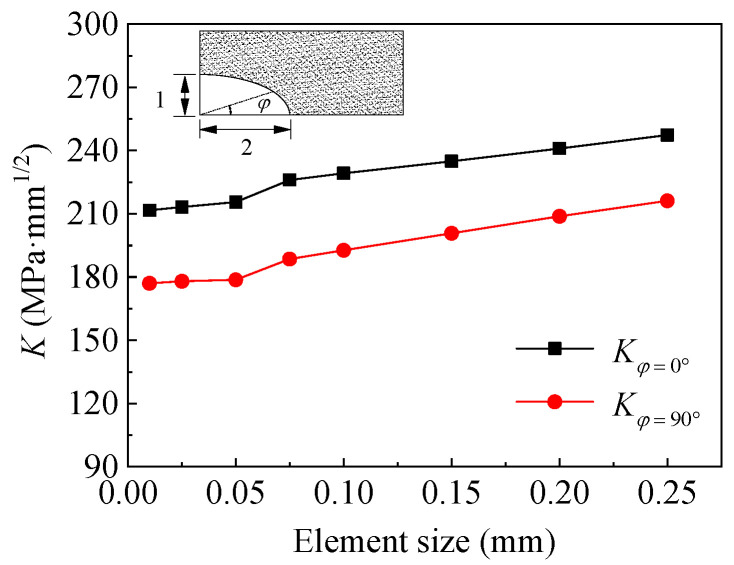
Variation in *K*-value with respect to element size.

The accuracy of the SIF calculation is the key to predicting the fatigue crack propagation of the corroded steel plate strengthened with CFRP plates. The main factor affecting *K*-value is the element size before and after crack front. To assure the feasibility and accuracy of the above finite element model and calculation method of SIF at crack tip, a mesh convergence study was carried out to ensure that the *K*-value at the crack tip of the corroded steel plate was convergent. Several element sizes of the finite element models were performed, and the variation in *K*-value with respect to element size is presented in Figure 14. As shown in Figure 14, the *K*-value is almost convergent for an element size of 0.05. In this study, the simulation models for the corroded steel plate strengthened with and without CFRP plates were constructed with an element size of 0.025.

**Figure 15:**
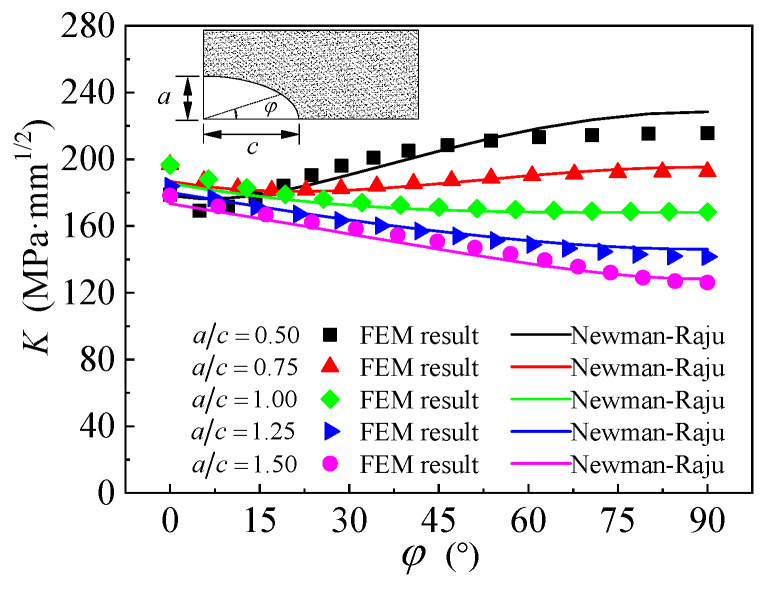
Comparison of *K*-values obtained by FEM and calculated adopting Newman-Raju formula.

To further verify the reliability of the numerical modeling method proposed in this paper, the *K*-value along the crack front of the equivalent initial semi-elliptical surface crack in the un-strengthened corroded steel plate was numerically analyzed and compared with the calculation result of the Newman-Raju formula [52]. Figure 15 illustrates the comparison of *K*-values obtained by FEM and calculated adopting Newman-Raju formula, where the thickness and width of the steel plate are 10 mm and 35 mm, respectively; the depths of all the semi-elliptical surface cracks are 1.0 mm, and the depth/width ratio are presented as 0.50, 0.75, 1.00, 1.25, and 1.50, respectively; the applied load is 50 kN. As shown in Figure 15, the *K*-values along the front of the semi-elliptical surface cracks with different depth/width ratios obtained by FEM in this study present an excellent correlation with those calculated adopting the Newman-Raju formula. The average error of the numerical results with respect to the analytical value is only 1.75%. This indicates that the finite element model and calculation method of SIF proposed in this paper can be used to calculate the SIF values of semi-elliptical surface cracks in corroded steel plate strengthened with CFRP plates with reasonable accuracy.

To further verify the reliability of the fatigue crack propagation prediction method of corroded steel plate strengthened with CFRP plates proposed in this paper, the fatigue test result represented in the authors’ recent study [14] is adopted for comparison with the prediction results in this paper. The prediction results of the fatigue crack propagation life of the fatigue tested specimens are listed in Table 2, where $N_{\exp}$ is the experimental results, $2c_{0,\max}$ and $a_{0,\max}$ are the width and depth of the critical rust pit (i.e., the deepest rust pit), respectively. $2c_{0,\mathrm{ave}}$ is the average width of all the rust pits on the corroded steel surface. The dimensions of the equivalent initial semi-elliptical surface crack include two kinds of constitution, where the width and depth of the critical rust pit (i.e., the deepest rust pit) are taken as the equivalent dimension of the type I crack, the maximum depth and the average width of the rust pits are taken as the equivalent dimension of the type II crack. $N_{p1}$ and $N_{p2}$ are the prediction values of the fatigue crack propagation life corresponding to type I equivalent crack and type II equivalent crack, respectively. It can be found from Table 2 that although the difference between the dimensions of these two types of equivalent cracks is not significant, where the depth of the two equivalent initial cracks is the same and the width difference is just approximately 1.0 mm, the prediction values of the fatigue crack propagation life corresponding to these two types of equivalent cracks are quite different. This indicates that the equivalent initial crack size has a great influence on the crack propagation life of the corroded steel plate strengthened with CFRP plates.

**Figure 16:**
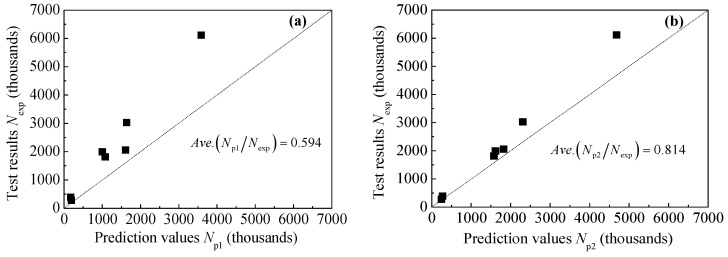
Comparison between prediction values and test results: (**a**) $N_{p1}$ versus $N_{\exp}$ (**b**) $N_{p2}$ versus $N_{\exp}$.

The appropriate equivalent initial crack is the key to fatigue life prediction. As mentioned in Section 5, a unified understanding of the identification and equivalent of the critical rust pits in the fatigue crack propagation prediction of the corroded metal plate has not been formed. Comparing the prediction values with the test results, it is considered to be more reasonable to calculate the fatigue propagation life of the corroded steel plate strengthened with CFRP plates by using the equivalent dimensions of type II crack, i.e., the maximum depth and the average width of the rust pits are suggested to be taken as the equivalent dimensions of the initial semi-elliptical surface crack. The main reasons may be as follows:

(1) The depth of the rust pits is the most important factor determining the initiation and propagation of fatigue crack, and the idea that the maximum pit depth is taken as the depth of the equivalent crack has been verified in many literatures [45,47]. The average pit width of the rust pits is equivalent to the initial crack width, which makes the width more representative.
(2) The widths of these two types of cracks in Table 2 are greater than the corresponding crack depths. As for semi-ellipsoidal rust pits, when the pit width is greater than its depth, the maximum stress point appears at the bottom of the rust pit, and the smaller the pit width/depth ratio, the more serious the stress concentration of the rust pit [53]. By comparing the widths of these two types of equivalent cracks in Table 2, it can be found that the average width ($2c_{0,\mathrm{ave}}$) of every specimen is smaller than the width of the critical rust pit ($2c_{0,\max}$). From the perspective of stress concentration, the deep and narrow rust pit corresponding to the type II equivalent crack is more prone to fatigue crack initiation than the deep and wide rust pit which corresponds to the type I equivalent crack.
(3) From the perspective of calculation accuracy, the prediction values of fatigue crack propagation life corresponding to the type I equivalent crack are too conservative compared with the test results, whereas the prediction values of fatigue crack propagation life corresponding to type II equivalent crack are in better agreement with the test results. Figure 16 presents the comparison between the prediction values and test results. As presented in Figure 16, the average values of $N_{p1}/N_{\exp}$ and $N_{p2}/N_{\exp}$ are 0.595 and 0.814, respectively. Considering the discreteness of fatigue tests, the calculation accuracy corresponding to the type II equivalent crack is still satisfactory, and the prediction values are still safe compared to the test results, which is of great practical interest for engineering applications.

## 8. Parameter Analysis

To further investigate the influence of corrosion and CFRP strengthening on the fatigue behavior of the corroded steel plate strengthened with CFRP plates, the two-stage crack propagation model proposed in this study is applied to carry out a parameter analysis to study the effect of the weight-loss rate of the corroded steel plate, the equivalent initial crack size, the adhesive thickness, the stiffness and prestress level of CFRP plates on the SIF values at crack tip and the fatigue crack propagation of the corroded steel plate strengthened with CFRP plates. Unless otherwise specified, the maximum applied load of all the following parameter analysis models is 96 kN and the load ratio is 0.1.

### 8.1. Effect of Weight-Loss Rate of the Corroded Steel Plate

**Figure 17:**
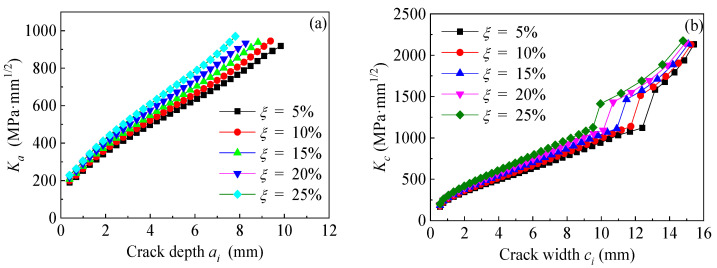
Effect of weight-loss rate of the corroded steel plate on the *K*-values at crack tip of the corroded steel plate strengthened with CFRP plates: (**a**) *K*_a_ vs. crack depth $a_{i}$ and (**b**) *K*_c_ vs. crack width $c_{i}$.

**Figure 18:**
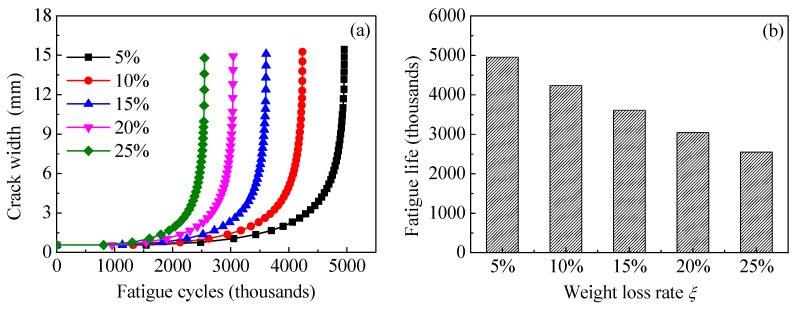
Effect of weight-loss rate on (**a**) the crack propagation curves and (**b**) the fatigue life of the corroded steel plate strengthened with CFRP plates.

The specimen C6-DS1-A in Table 2 is taken as the prototype structure to establish the models. All the dimensions and strengthening configurations of specimen C6-DS1-A remain unchanged except for the weight loss rates of the corroded steel plate, which are preset as 5%, 10%, 15%, 20% and 25%, respectively. Figure 17a,b depicts the effect of weight loss rate of the corroded steel plate on the values of *K*_a_ and *K*_c_ at crack tip of the corroded steel plate strengthened with CFRP plates, respectively, where *K*_a_ and *K*_c_ are the *K*-values corresponding to the deepest point and the widest point along the crack front, respectively. As shown in Figure 17, both the values of *K*_a_ and *K*_c_ increase with the increase in the crack size and the weight loss rate of the corroded steel plate. Furthermore, the larger the crack size, the more obvious the difference in the *K*-values caused by the increasing weight loss rate of the corroded steel plate. Figure 18a,b illustrate the effect of weight loss rate of the corroded steel plate on the crack propagation curves and fatigue life, respectively. As illustrated in Figure 18, the crack growth rate increases, whereas the fatigue life decreases with the increase in the weight loss rate of the corroded steel plate. When the weight loss rate of the corroded steel plate increases from 5% to 25%, the fatigue crack propagation life of the corroded steel plate strengthened with CFRP plates decreases from 4.96 million to 2.55 million with a reduction of about 50%.

### 8.2. Effect of Equivalent Initial Crack Size

**Figure 19:**
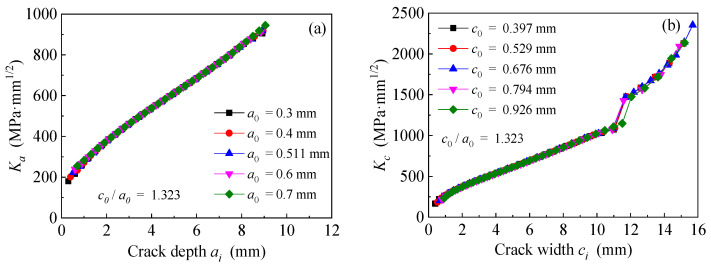
Effect of equivalent initial crack size on the *K*-values at crack tip of the corroded steel plate strengthened with CFRP plates: (**a**) *K*_a_ vs. crack depth $a_{i}$, and (**b**) *K*_c_ vs. crack width $c_{i}$.

The models are established based on the dimensions and strengthening configurations of specimen C9-DS1-A. The depth/width ratio of equivalent initial cracks remain unchanged, whereas the equivalent initial crack depths $a_{0}$ are preset as 0.3 mm, 0.4 mm, 0.511 mm (i.e., specimen C9-DS1-A), 0.6 mm and 0.7 mm, respectively. Figure 19a,b present the effect of equivalent initial crack size on the value of *K*_a_ and *K*_c_ at crack tip of corroded steel plate strengthened with CFRP plate, respectively. As presented in Figure 19, when the depth/width ratio of equivalent initial cracks remains unchanged, the initial crack depth only presents an effect on the *K*-values at the crack tip at the initial stage of crack propagation, and it has no influence on the subsequent variation law of SIF values versus crack growth size.

**Figure 20:**
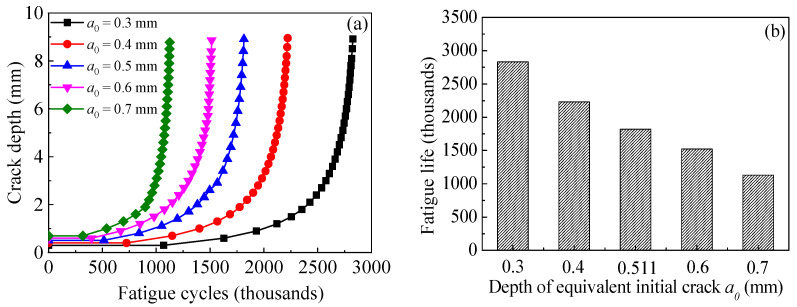
Effect of equivalent initial crack size on (**a**) the crack propagation curves and (**b**) the fatigue life of the corroded steel plate strengthened with CFRP plates.

Figure 20a,b shows the effect of equivalent initial crack size on the crack propagation curves and the fatigue life of the corroded steel plate strengthened with CFRP plates, respectively. As shown in Figure 20, the initial crack size has a significant effect on the fatigue crack propagation. The larger the initial crack size, the higher the crack growth rate and the lower the fatigue crack propagation life. Interestingly, when the depth of equivalent initial crack increases from 0.3 mm to 0.4 mm with an increment of 0.1 mm, the corresponding fatigue crack propagation life decreases by 605 thousand cycles, while when the depth of equivalent initial crack increases from 0.6 mm to 0.7 mm with the same increment of 0.1 mm, the decrement in the corresponding fatigue crack propagation life is merely 393 thousand cycles. This confirms that a larger part of the crack propagation life is consumed by crack growth of the smaller crack size. The smaller the initial crack size, the more sensitive the crack propagation life to the variation in equivalent initial crack size. It can also be found from Figure 20 that when the depth of the equivalent initial cracks increases from 0.3 mm to 0.7 mm with an increase of merely 0.4 mm, the corresponding fatigue crack propagation life decreases by more than 60%. This indicates that when the CFRP strengthening system is applied to improve the fatigue performance of the corroded steel structures, it should be carried out when the dimensions of the rust pits or the cracks of the corroded steel plate are still small, and early repair is suggested in order to achieve the best fatigue life extension of the corroded steel structures.

### 8.3. Effect of Adhesive Thickness

**Figure 21:**
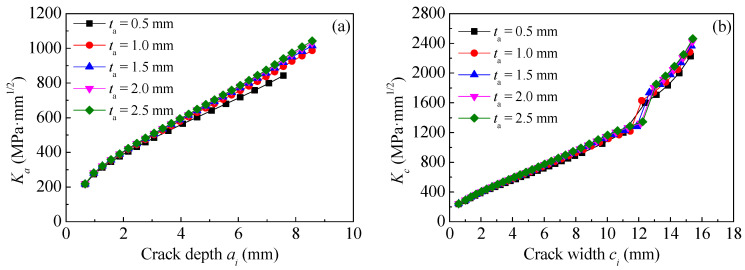
Effect of adhesive thickness on the *K*-values at crack tip of the corroded steel plate strengthened with CFRP plates: (**a**) *K*_a_ vs. crack depth $a_{i}$, and (**b**) *K*_c_ vs. crack width $c_{i}$.

The specimen C15-DS1-A in Table 2 is taken as the prototype structure used to establish the models. All the dimensions and strengthening configurations of specimen C15-DS1-A remain unchanged, except for the adhesive thickness, which is preset as 0.5 mm (i.e., specimen C15-DS1-A), 1.0 mm, 1.5 mm, 2.0 mm and 2.5mm, respectively. Figure 21a,b illustrate the effect of adhesive thickness on the value of *K*_a_ and *K*_c_ at crack tip of corroded steel plate strengthened with CFRP plate, respectively. As shown in Figure 21, the values of *K*_a_ and *K*_c_ present a very small increasing trend with the increase in the adhesive thickness. To interpret this phenomenon, the greater the adhesive thickness, the smaller the shear stiffness and the lower the load transfer efficiency of the interface between corroded steel plate and CFRP plates, and hence, the weaker the “bridging effect” of CFRP plate on restraining crack opening, and the larger the stress intensity factor at the crack tip. Furthermore, the reduction in load transfer efficiency caused by the increase in adhesive thickness will only present a limited impact on a limited range at the end of CFRP plates, since the end anchorage measures are taken for all the specimens in FEMs and the bonding length of the CFRP plates in the models is greater than the effective bonding length of the bond interface. The increasing trend of the *K*-values with the increase in the adhesive thickness is not obvious.

**Figure 22:**
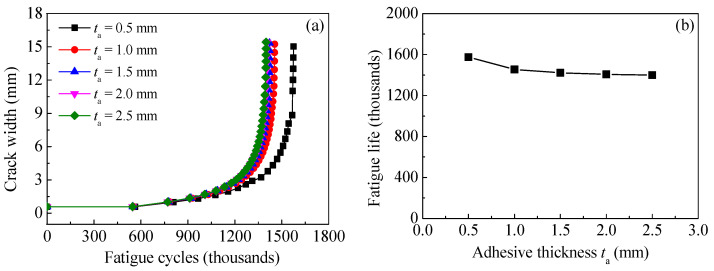
Effect of adhesive thickness on (**a**) the crack propagation curves and (**b**) the fatigue life of the corroded steel plate strengthened with CFRP plates.

Figure 22a,b present the effect of adhesive thickness on the crack propagation curves and the fatigue life of the corroded steel plate strengthened with CFRP plates, respectively. As shown in Figure 22, the greater the adhesive thickness, the higher the crack growth rate, and the lower the crack propagation life. Interestingly, the reduction in fatigue life tended to decrease with the increase in adhesive thickness. For example, when the adhesive thickness increased from 0.5 mm to 1.0 mm with an increment of 0.5 mm, the corresponding fatigue life decreased from 1,574,935 cycles to 1,454,737 cycles with a reduction of about 120,200 cycles, while when the adhesive thickness continues to increase from 2.0 mm to 2.5 mm, the corresponding reduction of fatigue life was only 7400 cycles. This indicates that the influence of adhesive thickness on the fatigue life is limited and convergent.

### 8.4. Effect of Stiffness of CFRP Plate

**Figure 23:**
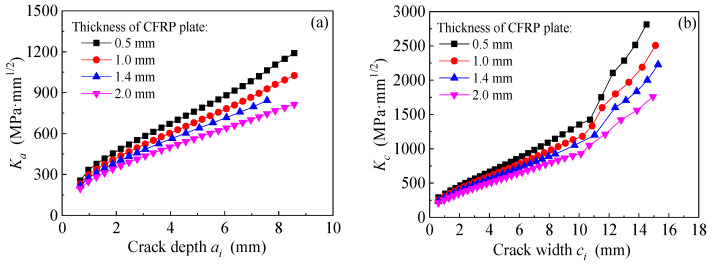
Effect of stiffness of CFRP plate on the *K*-values at crack tip of the corroded steel plate strengthened with CFRP plates: (**a**) *K*_a_ vs. crack depth $a_{i}$ and (**b**) *K*_c_ vs. crack width $c_{i}$.

The models are established based on the dimensions and strengthening configurations of specimen C15-DS1-A. The thickness of the external bonded CFRP plates which present the strengthening stiffness are preset as 0.5 mm, 1.0 mm, 1.4 mm (i.e., specimen C15-DS1-A) and 2.0 mm, respectively. Figure 23a,b depict the effect of strengthening stiffness of CFRP plate on the values of *K*_a_ and *K*_c_ at the crack tip of corroded steel plate strengthened with CFRP plate, respectively. As shown in Figure 23, as the thickness of CFRP plate increases from 0.5 mm to 1.0, 1.4 and 2.0 mm, the values of *K*_a_ and *K*_c_ which correspond to the same crack size, decrease continuously, and the decrement of *K*-values caused by the increasing thickness of CFRP plate becomes more and more significant with the increases in crack size. Interpretations made based on Figure 23 may be summarized as follows: the increase in the thickness of CFRP plate and the crack propagation size lead to the increase in the strengthening stiffness ratio, which is expressed as the ratio of the CFRP plate stiffness to the residual stiffness of the steel plate corresponding to the crack propagation section. The “bridging effect” of CFRP plate on restraining the crack opening becomes more and more obvious, resulting in the continuous reduction of the *K*-values at the crack tip.

**Figure 24:**
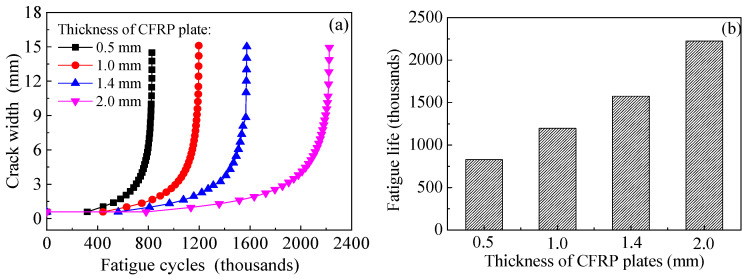
Effect of stiffness of CFRP plate on (**a**) the crack propagation curves and (**b**) the fatigue life of the corroded steel plate strengthened with CFRP plates.

Figure 24a,b illustrate the effect of stiffness of CFRP plates on the crack propagation curves and the fatigue life of the corroded steel plate strengthened with CFRP plates, respectively. As shown in Figure 24, with the increase in the strengthening stiffness of CFRP plates, the crack propagation rate decreases, and the fatigue life increases. When the thickness of CFRP plate increases from 0.5 mm to 1.0 mm, 1.4 mm and 2.0 mm, it leads to fatigue life extension of 1.44 times, 1.90 times and 2.69 times, respectively. This indicates that the fatigue strengthening effect of the corroded steel plate with external patched CFRP plates can be significantly improved with the increasing thickness (stiffness) of the CFRP plates.

### 8.5. Effect of Prestress Level of CFRP Plate

The models are established based on the dimensions and strengthening configurations of specimen C15-DS1-PS1-A. The prestress level of CFRP plate, which is defined as the ratio of the prestress to the tensile strength of the CFRP plate, is preset as 0%, 4.5% (i.e., specimen C15-DS1-PS1-A), 10%, 15% and 20%, respectively. The temperature reduction method is adopted to apply prestress to the CFRP plates, and the corresponding temperature loads, according to Equation (25), are 0 °C, −65.45 °C, −145.45 °C, −218.18 °C and −290.91 °C, respectively.

**Figure 25:**
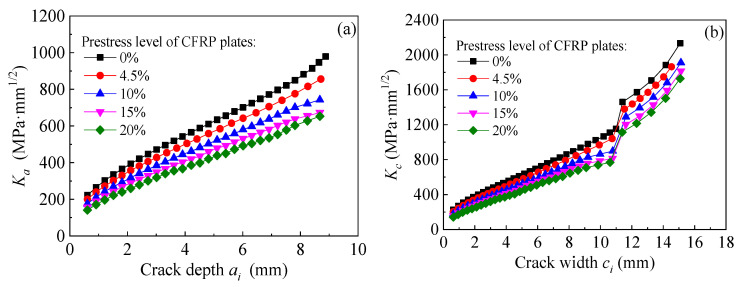
Effect of prestress level of CFRP plate on the *K*-values at crack tip of the corroded steel plate strengthened with CFRP plates: (**a**) *K*_a_ vs. crack depth $a_{i}$ and (**b**) *K*_c_ vs. crack width $c_{i}$.

Figure 25a,b present the effect of prestress level of CFRP plate on the values of *K*_a_ and *K*_c_ at the crack tip of the corroded steel plate strengthened with CFRP plates. As presented in Figure 25, both the values of *K*_a_ and *K*_c_ at the crack tip can be significantly reduced by applying pretension stress to the CFRP plates, and the *K*-values decrease continuously with the increasing prestress of the CFRP plates. The larger the crack size, the greater the reduction in *K*-values.

**Figure 26:**
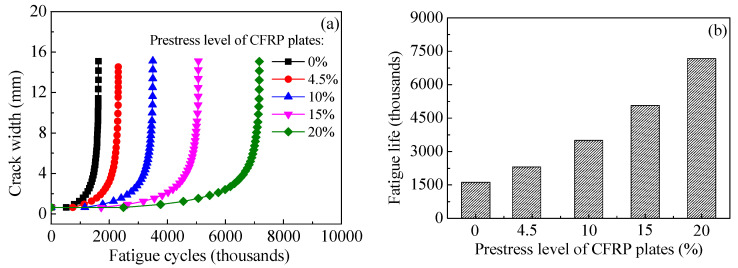
Effect of prestress level of CFRP plate on (**a**) the crack propagation curves and (**b**) the fatigue life of the corroded steel plate strengthened with CFRP plates.

Figure 26a,b show the effect of prestress level of CFRP plate on the crack propagation curves and the fatigue life of the corroded steel plate strengthened with CFRP plates, respectively. It can be found from Figure 26 that the higher the CFRP plate prestress level, the lower the crack propagation rate and the higher the fatigue life of the corroded steel plate strengthened with CFRP plates. As for the specimens with the same strengthening stiffness, the corresponding fatigue life of the specimen strengthened with a prestress level of 20% is 4.42 times greater than that of the specimen without prestressing force, indicating that application of prestressing can significantly improve the utilization rate of CFRP materials and the fatigue strengthening effect of the corroded steel plate. What should be pointed out is that the higher the prestress level of the CFRP plate, the higher the requirements for the interface bonding performance and anchorage measures. Good bonding and anchorage performance are the premise of the prestressed CFRP strengthening system.

## 9. Conclusions

In the present study, a new two-stage fatigue crack propagation prediction model for the corroded steel plate strengthened with CFRP plates was proposed; moreover, the identification of critical rust pits and the equivalent method of initial cracks, and the calculation method of stress intensity factor (SIF) values at the crack tip were established. The predictive analysis of the fatigue crack propagation of corroded steel plate strengthened with CFRP plates were conducted, and comparison between prediction values and fatigue test results was adopted to verify the reliability of the fatigue crack propagation prediction. Finally, the proposed two-stage crack propagation model was applied to carry out the parameter analysis to investigate the effect of weight loss rate, equivalent initial crack size, adhesive thickness, CFRP stiffness and CFRP prestress level on the fatigue crack propagation of the corroded steel plate strengthened with CFRP plates. Some conclusions could be made based on this research program:

(1) The proposed two-stage fatigue crack propagation prediction model could be used to predict the fatigue crack propagation process of the corroded steel plate strengthened with CFRP plates with reasonable accuracy.
(2) The maximum depth and the average width of the rust pits were suggested as the equivalent dimensions of the initial semi-elliptical surface crack for the fatigue crack propagation prediction of corroded steel plate strengthened with CFRP plates.
(3) As for the corroded steel plates double-side patched with CFRP plates, the maximum value of SIF gradually shifted from the deepest point of the crack to the surface of the steel plate during the partial-through crack propagation stage, whereas the SIF values during the through crack propagation stage were symmetrically distributed along the thickness direction of the steel plate, and the maximum value of SIF was located in the middle of the plate thickness.
(4) Increasing the weight loss rate of the corroded steel plate, the initial crack size or the adhesive thickness would accelerate the crack growth and reduce the fatigue life, while increasing the stiffness or prestress level of CFRP plate would significantly reduce the crack growth rate and increase the fatigue life. The smaller the initial crack size, the more sensitive the crack propagation life was to the variations in equivalent initial crack size. The influence of adhesive thickness on the fatigue life was limited and convergent, and the application of prestressing could significantly improve the utilization rate of CFRP materials and the fatigue strengthening effect of the corroded steel plate.

## Figures and Tables

**Table 1:** Primary mechanical properties of the materials adopted in the fatigue test.

Material	Specification	Thickness/mm	Tensile Modulus of Elasticity/GPa	Yield Strength/MPa	Tensile Strength/MPa	Elongation at Break %
CFRP plate	CFP-514	1.4	165 ^a^	N/A	2400 ^a^	1.61 ^a^
	CFP-520	2.0	165 ^a^	N/A	2400 ^a^	1.61 ^a^
Adhesive	Sikadur-30CN	N/A	5.3	N/A	41.75	1.13
Steel plate	Q235B	10.75 ^b^	181.9	275.6	421.2	20.8

^a^ According to the manufacturer’s instructions. ^b^ Thickness of steel plate cut from the flange of the uncorroded H beams.

**Table 2:** Results summary of the fatigue test and fatigue crack propagation prediction.

Specimens No.	*C*_d_ /Month	*ξ* /%	Strengthening Configurations	CFRP Prestress Level	CFRP Specification	$N_{\exp}$ /×10^3^	$2c_{0,\max}$ /μm	$a_{0,\max}$ /μm	$2c_{0,\mathrm{ave}}$ /μm	$N_{p1}$ /×10^3^	$N_{p2}$ /×10^3^	$\frac{N_{p1}}{N_{\exp}}\;$	$\frac{N_{p2}}{N_{\exp}}$
C6-DS1-A	6	9.16	Case D	-	CFP-514	6115.896	2200	390	1124	3584.865	4684.312	0.586	0.766
C6-U-S1	6	9.59	Case A	-	-	332.413	2400	356	1351	-	-	-	-
C9-DS1-A	9	13.25	Case D	-	CFP-514	2057.247	1700	511	1351	1607.245	1820.874	0.781	0.885
C9-U-S1	9	13.54	Case A	-	-	220.484	2200	441	1159	-	-	-	-
C15-DS2-A	15	16.38	Case D	-	CFP-520	17,240 *	3300	383	1275	1691.626	3124.658	-	-
C15-DS1-PS-A	15	15.27	Case D	4.5%	CFP-514	3025.477	2400	612	1264	1634.713	2309.643	0.540	0.763
C15-DS1-A	15	16.99	Case D	-	CFP-514	1808.695	2300	667	1166	1078.600	1574.935	0.596	0.871
C15-DS1	15	16.26	Case B	-	-	280.840	2200	553	1211	195,232	248.567	0.695	0.885
C15-SS1-A	15	16.42	Case C	-	CFP-514	389.139	1900	520	1216	178.099	277.928	0.458	0.714
C15-U-S1	15	16.52	Case A	-	-	202.164	2600	434	1199	-	-	-	-
C18-DS1-A	18	21.78	Case D	-	CFP-514	1995.725	3300	365	1229	1001.530	1617.287	0.502	0.810
C18-U-S1	18	21.19	Case A	-	-	175.414	3700	493	1097	-	-	-	-

Notes: *C*_d_ is corrosion duration; *ξ* is weight loss rate; $N_{\exp}$ is the experimental results of the fatigue life; $2c_{0,\max}$ and $a_{0,\max}$ are the width and depth of the critical rust pit (i.e., the deepest rust pit); $2c_{0,\mathrm{ave}}$ is the average width of the rust pits on the corroded steel surface; $N_{p1}$ and $N_{p2}$ are the prediction value of the fatigue crack propagation life corresponding to type I equivalent crack size and type II equivalent crack size, respectively. * As for specimen C15-DS2-A, no damage was found after 17 million fatigue cycles, and the test was stopped artificially.

**Table 3:** The whole process of the fatigue crack propagation prediction of specimen C9-DS1-A.

Analysis Step	$c_{i}$ /mm	$a_{i}$ /mm	$K_{c}$ /MPa·mm^1/2^	$K_{a}$ /MPa·mm^1/2^	$U\Delta K_{c}$ /MPa·mm^1/2^	$U\Delta K_{a}$ /MPa·mm^1/2^	$\Delta a_{i}$ /mm	$\Delta c_{i}$ /mm	$\Delta N_{i}$	$\sum_{i=1}^{i}\Delta N_{i}$
1	0.68	0.51	202.6	222.0	132.1	144.8	0.30	0.22	513,661	513,661
2	0.89	0.81	249.5	252.7	162.7	164.8	0.30	0.29	326,988	840,649
3	1.18	1.11	288.5	287.0	188.1	187.1	0.30	0.31	209,929	1,050,578
4	1.49	1.41	322.7	319.1	210.4	208.1	0.30	0.31	145,000	1,195,578
5	1.80	1.71	353.6	349.2	230.6	227.7	0.30	0.31	105,918	1,301,496
6	2.11	2.01	382.9	377.4	249.7	246.1	0.30	0.32	80,745	1,382,241
7	2.43	2.31	411.2	404.6	268.1	263.9	0.30	0.32	63,348	1,445,588
8	2.74	2.61	436.8	430.2	284.8	280.5	0.30	0.32	51,179	1,496,768
9	3.06	2.91	462.7	454.2	301.7	296.2	0.50	0.53	70,567	1,567,335
10	3.59	3.41	504.6	493.5	329.0	321.8	0.50	0.54	52,824	1,620,159
11	4.13	3.91	546.5	531.3	356.3	346.4	0.50	0.55	40,856	1,661,015
12	4.69	4.41	589.1	568.4	384.1	370.6	0.50	0.57	32,287	1,693,302
13	5.25	4.91	633.3	605.1	413.0	394.6	0.50	0.59	25,952	1,719,254
14	5.84	5.41	679.5	641.8	443.1	418.5	0.50	0.61	21,142	1,740,396
15	6.45	5.91	728.9	678.3	475.3	442.3	0.50	0.64	17,434	1,757,830
16	7.09	6.41	781.3	715.5	509.5	466.5	0.50	0.68	14,469	1,772,299
17	7.77	6.91	837.3	753.8	546.0	491.5	0.50	0.72	12,065	1,784,364
18	8.49	7.41	898.5	794.0	585.9	517.7	0.50	0.77	10,065	1,794,430
19	9.26	7.91	965.2	836.9	629.4	545.7	0.50	0.82	8377	1,802,806
20	10.08	8.41	1023.9	877.2	667.7	572.0	0.50	0.86	7111	1,809,917
21	10.94	8.91	1085.9	918.2	708.1	598.7	0.42	0.75	5033	1,814,950
22	11.69	9.33	1469.7	-	958.3	-	-	0.50	1176	1,816,126
23	12.19	-	1530.4	-	997.9	-	-	0.50	1021	1,817,147
24	12.69	-	1596.6	-	1041.0	-	-	0.50	881	1,818,028
25	13.19	-	1672.8	-	1090.8	-	-	0.50	749	1,818,777
26	13.69	-	1759.4	-	1147.2	-	-	0.50	628	1,819,405
27	14.19	-	1862.9	-	1214.7	-	-	0.50	514	1,819,919
28	14.69	-	1986.7	-	1295.4	-	-	0.50	411	1,820,331
29	15.19	-	2143.0	-	1397.4	-	-	0.50	316	1,820,646
30	15.69	-	2353.9	-	1534.9	-	-	0.50	228	1,820,874

Note: The maximum depth and the average width of the rust pits on the surface of the corroded steel plate are taken as the equivalent dimension of the initial semi-elliptical surface crack in step 1. Step 1~step 21 belong to the partial-through crack propagation stage, and step 22~step 30 belong to the through crack propagation stage.

## Data Availability

The data presented in this study are available on request from the corresponding author.
